# Retention of knowledge and clinical competence among Ugandan mid-level health providers 1 year after intensive clinical mentorship in TB and HIV management

**DOI:** 10.1186/s12960-021-00693-x

**Published:** 2021-12-09

**Authors:** Dan K. Senjovu, Sarah Naikoba, Pallen Mugabe, Damazo T. Kadengye, Carey McCarthy, Patricia L. Riley, Shona Dalal

**Affiliations:** 1 Infectious Diseases Institute, College of Health Sciences, Makerere University Kampala, Kampala, Uganda; 2 Centers for Disease Control and Prevention, Kampala, Uganda; 3 Centers for Disease Control and Prevention, Atlanta, GA United States of America

**Keywords:** Task sharing, Clinical mentorship, HIV, TB, Mid-level providers

## Abstract

**Introduction:**

Clinical mentorship is effective in improving knowledge and competence of health providers and may be a useful task sharing approach for improving antiretroviral therapy. However, the endurance of the effect of clinical mentorship is uncertain.

**Methods:**

The midlevel health providers who participated in a cluster-randomized trial of one-on-one, on-site, clinical mentorship in tuberculosis and HIV for 8 h a week, every 6 weeks over 9 months were followed to determine if the gains in knowledge and competence that occurred after the intervention were sustained 6- and 12-months post-intervention. In December 2014 and June 2015, their knowledge and clinical competence were respectively assessed using vignettes and a clinical observation tool of patient care. Multilevel mixed effects regression analysis was used to compare the differences in mean scores for knowledge and clinical competence between times 0, 1, 2, and 3 by arm.

**Results:**

At the end of the intervention phase of the trial, the mean gain in knowledge scores and clinical competence scores in the intervention arm was 13.4% (95% confidence interval ([CI]: 7.2, 19.6), and 27.8% (95% CI: 21.1, 34.5) respectively, with no changes seen in the control arm. Following the end of the intervention; knowledge mean scores in the intervention arm did not significantly decrease at 6 months (0.6% [95% CI − 1.4, 2.6]) or 12 months (− 2.8% [95% CI: − 5.9, 0.3]) while scores in the control arm significantly increased at 6 months (6.6% [95% CI: 4.4, 8.9]) and 12 months (7.9% [95% CI: 5.4, 10.5]). Also, no significant decrease in clinical competence mean scores for intervention arm was seen at 6 month (2.8% [95% CI: − 1.8, 7.5] and 12 months (3.7% [95% CI: − 2.4, 9.8]) while in the control arm, a significant increase was seen at 6 months (5.8% [95% CI: 1.2, 10.3] and 12 months (11.5% [95% CI: 7.6, 15.5]).

**Conclusions:**

Mentees sustained the competence and knowledge gained after the intervention for a period of one year. Although, there was an increase in knowledge in the control group over the follow-up period, MLP in the intervention arm experienced earlier and sustained gains. One-on-one clinical mentorship should be scaled-up as a task-sharing approach to improve clinical care.

*Trial Registration* The study received ethics approvals from 3 institutions—the US Centers for Disease Control and Prevention Institutional Review Board (USA), the Institutional Review Board “JCRC’s HIV/AIDS Research Committee” IRB#1-IRB00001515 with Federal Wide Assurance number (FWA00009772) based in Kampala and the Uganda National Council of Science and Technology (Uganda) which approves all scientific protocols to be implemented in Uganda.

## Background

In September 2015, the United Nations made a commitment to end the epidemics of AIDS, tuberculosis, malaria and neglected tropical diseases by 2030. However, progress to end the epidemic in the most affected countries in sub-Saharan Africa is limited by the number of qualified health professionals [1], necessitating significant investments in the health workforce to reach targets set out by the 2015 UN Sustainable Development Goals by 2030 [2]. There is a need to increase the number of trained and competent health care workers. This can be accomplished in part through the World Health Organization (WHO) guidance on task shifting, whereby clinical tasks that were previously undertaken by physicians are taken on by mid-level health providers (MLP) [3].

MLPs can be defined as different cadres of health workers such as clinical officers, registered nurses, and registered midwives in different settings. In Uganda, all three of these categories of MLPs with diploma-level training are authorized to prescribe ART and TB medication, a role that was previously performed by doctors [4]. Clinical officers, unlike the registered nurses and midwives, are also allowed to conduct minor surgery; however, they are not trained to conduct deliveries. A key to task sharing is the need to ensure that quality of care is maintained among health workers that take on the additional clinical tasks that come with task sharing, which can be accomplished in part through clinical mentorship. Clinical mentoring is one approach of knowledge translation that uses social influence through interpersonal interactions to increase clinical knowledge and uptake of evidence-based practices [5, 6]. However, the methods of mentorship remain highly variable and there is scant evidence on which methods result in sustained knowledge and competence [7–9].

Uganda is a country with a high burden of both HIV and tuberculosis (TB), and suffers from a critical shortage of health workers, with a ratio of 0.093 physicians per 1000 people [10]. Uganda has endorsed on-site, clinical mentorship of MLP to develop their skills in providing high-quality HIV and TB treatment, and has published a pocket reference book for clinician mentors which focuses primarily on HIV management [11]. In order to improve clinical mentorship in public health facilities, it is essential that the most effective and sustainable knowledge and competence diffusion approaches [12–14] are identified and disseminated. A cluster-randomized trial described elsewhere [15] demonstrated the effectiveness of a one-on-one, on-site, clinical mentorship program on individual MLP knowledge and competence. This follow-up study, continues from the end of the intervention of the cluster-randomized trial and reports on follow up 6 and 12 months after the intervention period to determine if the gains seen were sustained once mentored providers returned to their routine practice.

## Methods

We describe 12-month observational study on knowledge, competence and clinical practice among MLP that completed a cluster-randomized trial of one-on-one clinical mentoring to determine if their gains in knowledge and clinical competence were sustained [16, 17] 6 and 12 months after the intervention ended. The detailed methods for the cluster-randomized trial are described elsewhere [15] but briefly, the intervention comprised of one-on-one, on-site clinical mentoring of MLP on HIV and TB care from a trained, randomly assigned mentor for 8 h a week, every 6 weeks, over a nine-month period at each of five intervention sites; no intervention or educational materials were provided in control sites. In Uganda, MLP typically do not receive regular training but may participate in short off-site training on specific topics or during dissemination of new or revised guidelines. The mentorship intervention package in this study was designed to increase knowledge, stimulate critical thinking, and improve patient management.

Eligible MLP were clinical officers, registered nurses, or registered midwives with diploma-level training (i.e., ≥ 3 years post-secondary school education), with 80% of workload dedicated to clinical management of TB and HIV. Study sites were Health Center level IV facilities, which were until recently, the lowest level at which the initiation of treatment, and follow up of TB and adult HIV patients was allowed. To be included in the study, a site was required to have a minimum of four MLP, and not be involved in the implementation of a similar intervention. Each MLP was paired with two mentors. The first, selected from the Infectious Diseases Institute (IDI) were clinical officers with at least 4 years of relevant clinical experience, expertise in HIV/ AIDS and TB care, and training in facilitation and mentoring coaching skills. The second mentor was from the district health system, and was selected in collaboration with the District Health Officer to facilitate continuity; these mentors had similar qualifications to the IDI-based mentor. Mentoring sessions covered specific scheduled topics but mentees were also able to discuss clinical cases and other questions or challenges that they wished to confer about. Mentors were available by phone during times that they were not on-site and could arrange meetings to discuss the challenges that mentees encountered.

Written informed consent was obtained from all MLP before enrollment in the study; no financial or material compensation was provided. Study participants had the right to opt out of the study at any time. A total of 40 MLP were randomly selected for enrollment (4 MLP at each of the five intervention and five control facilities), of which 39 (98%) were assessed for knowledge and competence at baseline (“Time 0”) and end of intervention (“Time 1”).

**Fig. 1:**
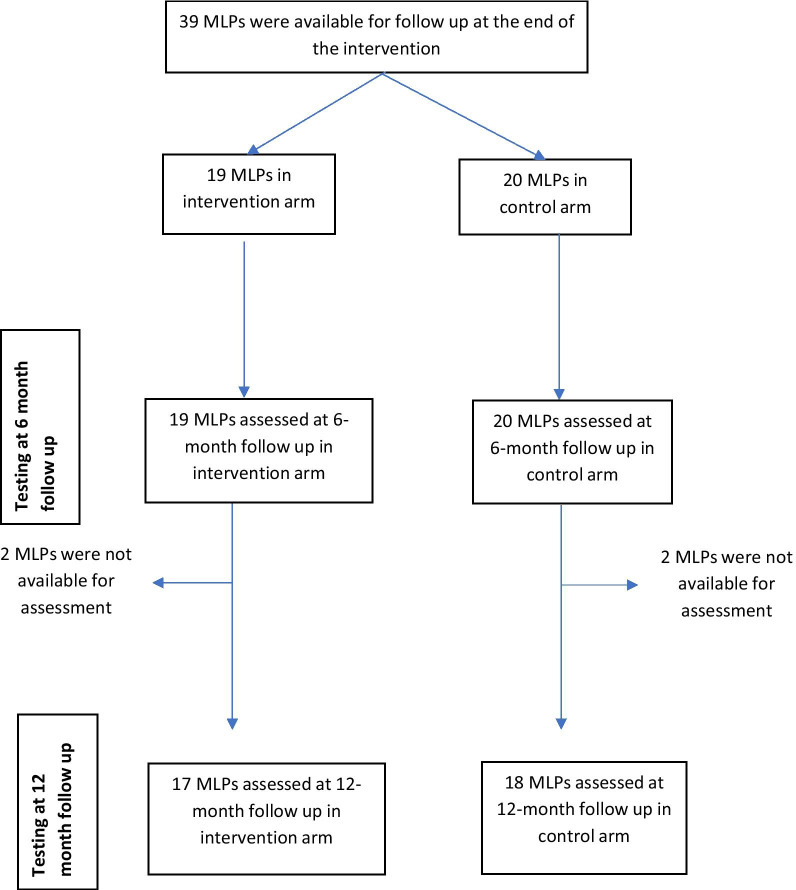
Timeline of MLP participation, follow up and testing

All the 39 MLP that completed the cluster-randomized trial were observed for one year to establish how the knowledge and competence they gained by the end of intervention varied. During the follow-up period, no clinical mentorship was provided to either the intervention arm or the control arm, but monthly monitoring was done to track study participants’ involvement in HIV and TB services. Refer to the CONSORT diagram in Fig. 1 for the timeline of MLP participation and Table 1 for characteristics of participants.

**Table 1:** Demographic and professional characteristics of mid-level providers^†^ and clinic characteristics by study arm

Characteristics	Control arm				Intervention arm			
	Baseline	End of intervention	6-month follow up	12 month follow up	Baseline	End of intervention	6 month follow up	12 month follow up
	Time 0	Time 1	Time 2	Time 3	Time 0	Time 1	Time 2	Time 3
	(N = 20)	(N = 20)	(N = 20)	(N = 18)	(N = 20)	(N = 19)	(N = 19)	(N = 17)
Sex (n [%])								
Female	15 (75)	15 (75)	15 (75)	14 (78)	16 (80)	15 (79)	15 (79)	14 (82)
Male	5 (25)	5 (25)	5 (25)	4 (22)	4 (20)	4 (21)	4 (21)	3 (18)
Professional Cadres (n [%])								
Clinical Officers	5 (25)	5 (25)	5 (25)	4 (22)	7 (35)	6 (32)	6 (32)	6 (35)
Registered nurses/midwives	15 (75)	15 (75)	15 (75)	14 (78)	13 (65)	13 (68)	13 (68)	11 (65)
Age (years), Mean (SD)	40 (11)	40 (11)	40 (11)	41 (12)	38 (8)	38 (8)	38 (8)	39 (8)
Years in practice, median (IQR)	4.5 (1.5, 7.5)	3.0 (1.5, 7.0)	3.0 (1.5, 7.0)	3.0 (1.5, 7.0)	3.0 (1.4, 10.0)	3.0 (1.4, 10.0)	3.0 (1.4, 10.0)	4.0 (1.4, 10.0)
Monthly outpatient attendances, Mean (SD)	1405 (455.6)	1405 (455.6)	1405 (455.6)	1387 (478.2)	1828 (716.0)	1846 (731.0)	1846 (731.0)	1914 (744.0)
Monthly active HIV patients, Mean (SD)	233.4 (84.2)	233.4 (84.2)	233.4 (84.2)	230.3 (88.4)	348.9 (131.3)	342.4 (131.5)	342.4 (131.)	337.2 (138.5)

^†^Mid-level providers refer to clinical officers, registered nurses, and midwives, and exclude medical officers

The 6-month (‘Time 2’) follow-up assessment for MLP knowledge and competence was conducted in December 2014 while the 12-month (‘Time 3’) follow-up assessment was conducted in June 2015. The same format of similar (but not the same) vignettes [18, 19] and standardized clinical observations used at Time 0 and Time 1 were used during Time 2 and Time 3 assessments. Knowledge assessments were conducted using four vignettes for each MLP that mimicked a HIV and/or TB patient visit. Knowledge areas assessed included: obtaining a clinical history, physical examination, ordering and interpreting laboratory investigations, making a diagnosis, treatment, and patient education/follow-up.

Clinical competence was assessed using the patient care observation tool, administered at the MLP work-stations. Each MLP was observed, assessed, and scored using a coded clinical observation checklist while managing each of four types of patients: 1- HIV patient not on ART, 2- HIV patient on ART, 3- TB patient on treatment, and 4- HIV patient with TB. The assessors observed how the MLP managed the patients from history taking, examination, to provision of treatment and patient education. In both knowledge and clinical competence measurements, clinical capability was determined and included in the results. The assessors were senior clinicians employed as tutors or examiners of training institutions for clinical officers and nurses. The assessors were blinded to the control or intervention arms. At the end of the study, MLP in the control group were provided with appropriate education and training resources such as participants’ training manuals used during the study and feedback on areas that could be strengthened.

### Data analysis

Observational data were entered into a pre-designed entry form in Epi-Data ver 3.1. Data cleaning and management were done on the anonymized dataset in Stata software (Stata release 11). Data from both the intervention trial and follow-up observational study (Time 0–3) were included in this analysis. For each arm, the mean difference between scores at 6-month (Time 2) and 12-month (Time 3) follow-up was compared to scores at baseline (Time 0) and the end of the trial (Time 1). A multilevel mixed effects regression analysis was used to compare the differences in MLP mean scores for knowledge and competence between times 0, 1, 2, and 3 by arm. This method was based on a two-time point repeated measures analysis treating sites as clusters to account for intra-site correlations. Stata release 11 (College Station, TX, USA) was used for all analyses.

### Ethics review

This study received human subjects ethical approval from the Joint Clinical Research Centre – Institutional Review Board (Uganda), the Uganda National Council of Science and Technology (Uganda) and the U.S. Centers for Disease Control and Prevention (CDC) Institutional Review Board (USA).

## Results

All 39 (100%) of the original trial MLP completed the 6-month (Time 2) follow-up, and 35 (90%) completed the 12-month (Time 3) follow-up (Fig. 1). Of the 35 that completed 12-months follow up, 18 (51%) were in the control arm and 17 (49%) were in the intervention arm.

### Retention of knowledge and competence

#### MLP Vignettes Knowledge Assessment

**Fig. 2:**
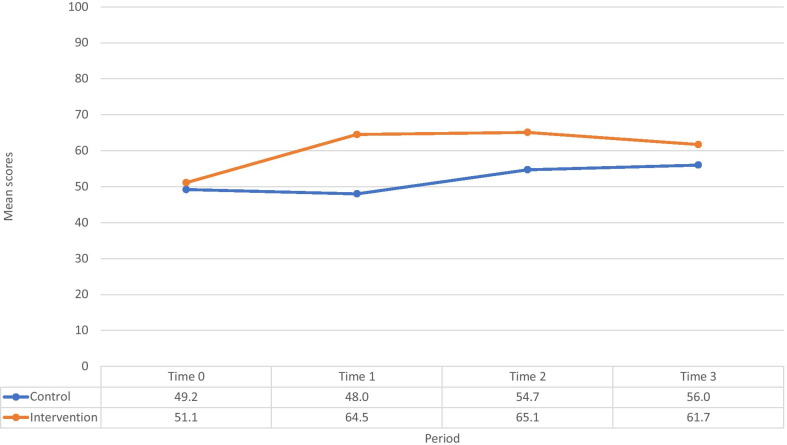
MLP knowledge measured with mean scores of vignettes across four time points

Figure 2 shows MLP knowledge assessment mean scores at Time 0, 1, 2, and 3. The average mean score for MLP knowledge in the intervention arm significantly improved from 51.1% at time 0 to 64.5% at time 1 (*p* < 0.001), and there was no significant change in knowledge in the control arm (49.2 (Time 0) to 48.0 (Time 1); *p* = 0.334). From the end of the trial (Time 1), mean knowledge scores in the intervention arm did not decrease at Time 2 (65.1%, *p* = 0.57) or at Time 3 (61.7%, *p* = 0.07). However, the mean knowledge score in the control arm increased from 48% at Time 1 to 54.7% (*p* < 0.001) at Time 2 and 56.0% at Time 3 (*p* < 0.001). When comparing the difference-in-difference in MLP mean scores at Time 0 to Time 2, there was a significant intervention effect (*p* = 0.037), however the difference was not significant at Time 3 due to the significant increases seen in the control arm (Table 2). Compared to the control arm, the rate of change in mean knowledge scores over time was lower in the intervention arm (*p* = 0.008) after the trial ended.

**Table 2:** Retention in knowledge and competence scores of mid-level providers in intervention and control study arms

Period	Mean change in KNOWLEDGE scores			Mean change in clinical competence scores		
	Intervention	Control	*p*-value**	Intervention	Control	*p*-value
Baseline to End of intervention:	13.4 (7.2, 19.6)	− 1.1 (− 3.5, 1.3)	< 0.001	27.8 (21.1, 34.5)	0.8 (− 3.4, 5.0)	< 0.001
Time 0 to Time 1, (95% CI*)						
Baseline to 6-month follow-up:	14.2 (8.3, 19.6)	5.5 (2.8, 8.2)	0.037	30.6 (25.7, 35.6)	6.6 (1.2, 12.0)	< 0.001
Time 0 to Time 2, (95% CI)						
Baseline to 12-month follow-up:	10.8 (5.0, 16.2)	6.8 (4.0, 9.7)	0.352	30.0 (25.5, 34.6)	11.7 (6.8, 16.6)	< 0.001
Time 0 to Time 3, (95% CI)						
End of intervention to 6-month follow-up:	0.6 (− 1.4, 2.5)	6.6 (4.4, 8.9)	0.102	2.8 (− 1.8, 7.5)	5.8 (1.2, 10.3)	0.476
Time 1 to Time 2, (95% CI)						
End of intervention to 12-month follow up:	− 2.8 (− 5.9, 0.3)	7.9 (5.4, 10.5)	0.004	3.7 (− 2.4, 9.8)	11.5 (7.6, 15.5)	0.056
Time 1 to Time 3, (95% CI)						

*CI = confidence interval; **p-value comparing intervention to control arms

#### Mid-level practitioners clinical competence assessments

**Fig. 3:**
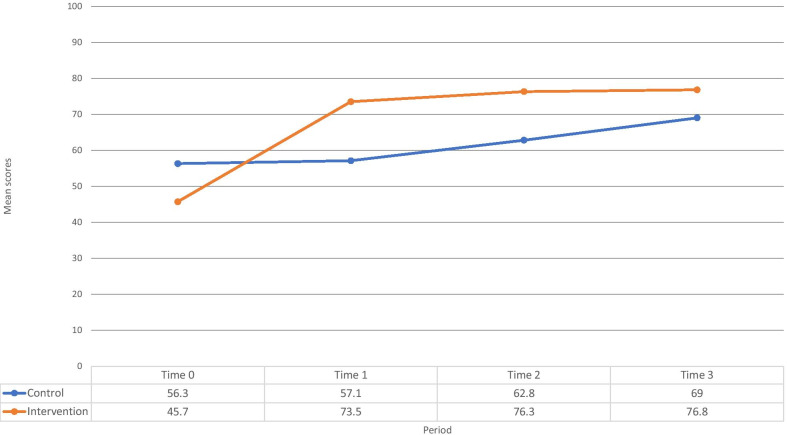
MLP clinical competence measured with mean scores in clinical observations across four time points

Figure 3 shows MLP clinical competence assessment scores at Time 0, 1, 2, and 3. The average mean score for MLP competency in the intervention arm significantly improved from 45.7% at time 0 to (73.5%) at time 1 ([95% CI: 21.1, 34.5]),) and there was no significant competency gain in the control arm (56.3% (Time 0) to 57.1% (Time 1); [95% CI: − 3.4, 5.0]). Compared to Time 1, the mean competency score did not change at Time 2 (76.3%, *p* = 0.215,) or at Time 3 (76.8%, *p* = 0.212) in the intervention arm. In the control arm, the mean competence score at Time 1 (57.1%) increased by Time 2 (62.8%, *p* = 0.015) and Time 3 (69.0%, *p* < 0.001). However, the interaction term between time and arm was not significant (*p* = 0.136). Over time, the rates of change in clinical scores were not statistically different for both arms. When comparing the difference-in-difference in MLP mean clinical competence scores at Time 0 to Time 2 and Time 3, statistically significant effects remained for the intervention arm (Table 2).

## Discussion

The findings of this study show that one-on-one on-site clinical mentorship of MLP resulted in improvement in knowledge and clinical competence that was sustained 6 and 12 months after the end of the mentorship program. Considering the fact that no additional support was provided to the intervention arm after the trial, this study’s findings showed a pattern different from most scientific domains that are characterized by progressive knowledge decay after initial acquisition of clinical science [20–23]. Sustaining gains in knowledge and clinical competence over time is the ultimate goal for any mentoring or teaching program, and is crucial to providing quality healthcare, particularly for conditions requiring long-term care such as TB and HIV.

With the urgent need to fill health workforce gaps in Uganda, training approaches that equip health care workers to take on more advanced task sharing while minimizing clinic disruption are critical from the perspectives of health systems, providers and patients; one-on-one mentoring is an approach that has the potential to meet these needs. MLP who received mentoring in the intervention arm had statistically higher scores in clinical competence than those in the control arm at both 6 and 12 month follow up, although some gains were also seen in the control arm. Similarly, the change in knowledge mean scores was significantly lower in the control arm than that of the intervention arm at 6 months follow-up. It was at 12-month follow-up that the difference in knowledge gain between the control arm and the intervention arm was not statistically significant. The gain in knowledge in the control arm could have stemmed from two study-related reasons. One; the vignettes, (which were used to assess similar clinical knowledge at all assessment points), are structured to encourage critical thinking of an individual [18, 19, 24–26] and could conceivably have resulted in improved understanding. Second, repeated test taking may have resulted in a “testing effect” whereby exposure to multiple similar tests may contribute to an increase in performance [27]. Improvement in knowledge can also occur through other means, including; by self-motivated individuals who read materials and prepare for assessment. However, although vignettes and the testing effect could have improved knowledge of MLP, it took a longer time for MLP in the control arm to reach similar levels of knowledge score of the intervention arm. Despite the increases seen in the control arm, the improvements in the intervention arm were early and sustained over time.

In post-mentoring feedback, MLP appreciated the mentoring relationship and reported that it improved their confidence in managing HIV and TB cases. This was demonstrated in the results from the cluster-randomized trial, which showed that mentored MLP handled 50% of HIV clinic consultations compared to 27% by their non-mentored peers, with an improvement in HIV and TB indicators at the facility level such as the proportions of patients who had been offered an HIV test [15]. An on-site mentorship approach thus had a cascade effect on clinical practice, patient care, and overall facility performance. Implementing a mentoring program such as this on a larger scale could improve the quality of clinical care with the existing health workforce, and also provide a means for continuing medical education for mentees. Moreover, engaging clinical officers who are currently employed through the government public health system as mentors, as was done in this study, enables future sustainability of mentoring relationships within existing systems with minimal additional costs.

The sample size of MLP used in this study limited the level of stratified analysis that could be done to assess knowledge and competence changes in specific sub-groups. Also, the study was implemented in a typical rural Uganda Health Centre IV’s and results may not be generalizable to health facilities in urban settings. Other limitations given in the trial paper potentially apply to this study.

## Conclusion

In conclusion, this study shows that one-on-one on-site mentorship led to sustained gains in clinical competence and knowledge among MLP 12 months after a mentorship program ended. Mentored MLP in the intervention arm experienced earlier and sustained gains compared to MLP in the control arm, with an encouraging post-intervention increase in knowledge seen in the control group. Maintaining quality patient care is vital to reaching new global targets to treat people living with HIV. Scaling up a one-on-one, on-site clinical mentoring program for MLP could enhance task sharing with minimal service disruptions, and provide continuing medical education for health care providers. Such an approach is one effective strategy to ease the critical health workforce shortage in resource-limited settings.

## Data Availability

The data that support the findings of this study are available from the Infectious Diseases Institute but restrictions apply to the availability of these data, which were used under license for the current study, and so are not publicly available. Data are however available from the authors upon reasonable request and with permission of the Infectious Diseases Institute.

## Abbreviations

CDC: Center for Disease Control and Prevention
HIV: Human Immunodeficiency Virus
JCRC: Joint Clinical Research Centre
MLP: Mid-level providers
TB: Tuberculosis
WHO: World Health Organization
